# Investigation of Lactones as Innovative Bio-Sourced Phase Change Materials for Latent Heat Storage

**DOI:** 10.3390/molecules24071300

**Published:** 2019-04-03

**Authors:** Rebecca Ravotti, Oliver Fellmann, Nicolas Lardon, Ludger J. Fischer, Anastasia Stamatiou, Jörg Worlitschek

**Affiliations:** Competence Centre Thermal Energy Storage (TES), Lucerne University of Applied Sciences and Arts, 6048 Horw, Switzerland; rebecca.ravotti@hslu.ch (R.R.); oliver.fellmann@hslu.ch (O.F.); nicolas.lardon@mr.mpg.de (N.L.); ludger.fischer@hslu.ch (L.J.F.); joerg.worlitschek@hslu.ch (J.W.)

**Keywords:** latent heat storage (LHS), phase change material (PCM), esters, lactones, cyclic esters, thermal energy storage

## Abstract

In the presented work, five bio-based and bio-degradable cyclic esters, i.e. lactones, have been investigated as possible phase change materials for applications in latent heat storage systems. Commercial natural lactones such as ε-caprolactone and γ-valerolactone were easily purchased through chemical suppliers, while 1,2-campholide, oxa-adamantanone and dibenzochromen-6-one were synthesized through Baeyer-Villiger oxidation. The compounds were characterized with respect to attenuated total reflectance spectroscopy and gas chromatography coupled with mass spectroscopy, in order to confirm their chemical structures and identity. Subsequently, thermogravimetric analysis and differential scanning calorimetry were used to measure the phase change temperatures, enthalpies of fusion, degradation temperatures, as well to estimate the degree of supercooling. The lactones showed a wide range of phase change temperatures from −40 °C to 290 °C, making them a high interest for both low and high temperature latent heat storage applications, given the lack of organic phase change materials covering phase change temperature ranges below 0 °C and above 80 °C. However, low enthalpies of fusion, high degrees of supercooling and thermal degradations at low temperatures were registered for all samples, rendering them unsuitable as phase change materials.

## 1. Introduction

Due to the increasing global energy consumption in addition to concerns related to the growing scarcity of fossil fuels and environmental degradation, energy efficiency and the integration of renewable energies in our energy systems are of utmost importance. Considering this, Thermal Energy Storage (TES) can be seen to provide plausible assistance to solving both issues. Latent Heat Storage (LHS) systems are increasingly gaining attention in the scientific community as a result of several advantages in comparison to current Sensible Heat Storage (SHS) systems, such as water or stone tanks [1,2,3,4]. In particular, they offer more compact systems and a high energy density, which is of crucial importance in urban environments due to space scarcity. As described in several sources, the LHS functioning principle relies on PCMs’ ability to store energy during specific phase transitions (e.g., solid-solid, solid-liquid), without directly changing the material’s temperature. In the case of solid-liquid PCMs, heat is absorbed during melting and released again upon crystallization of the material. To be classified as an attractive PCM, a material needs to primarily exhibit qualities including a congruent phase change over narrow temperature ranges, thermal stability over repeated cycles, high latent energy of phase transition (in the case of solid-liquid transition it is latent energy of fusion), and low supercooling [5,6,7,8]. In addition, PCMs should be cost-effective, present low to no toxicity and should ideally be bio-sourced and bio-degradable in order to reduce their environmental impact and help establish a more sustainable utilization of resources and energy. Until now, several inorganic (e.g., salt hydrates) and organic materials (e.g., paraffins and carboxylic acids) have already been tested as PCMs, with promising results in both categories. In particular, salt hydrates and paraffins are common choices as PCMs due to their low cost and ease of implementation, respectively. However, in addition to not being environmentally-friendly, they both display further disadvantages, including long term instability (segregation), high supercooling and corrosivity for salt hydrates, and high flammability in the case of paraffins. This makes them inadequate materials for high temperature applications and poses health and general safety risks. As a result, new categories of materials to be used as PCMs are currently being investigated. Amongst these, esters are becoming prevalent as a new alternative to traditional organic PCMs. Esters are chemical compounds formed by the combination of an acid (usually a carboxylic acid) with an alcohol with concomitant loss of a molecule of water. To date, despite being based on renewable feedstock in addition to being biodegradable and reportedly presenting interesting thermal characteristics, only a few research studies on esters in this context have been published. Of these, the majority focus in particular on esters derived from fatty acids. For example, Ravotti et al. [9,10], investigated the thermal properties in conjunction with the relationship between melting temperatures T_m_ and chemical structures of synthesized fatty esters derived from five carboxylic acids (myristic acid C_14_, palmitic acid C_16_, stearic acid C_18_, arachidic acid C*_20_* and behenic acid C_22_), each coupled with three alcohols of different chain lengths (methanol C_1_, and 1-decanol C_10_). As it emerged from the results of the study, all fatty esters showed high enthalpies of fusion, thermal stability over six consecutive heating and cooling cycles, and low to no supercooling, identifying them as possible new PCM candidates. Stamatiou et al. [11] investigated the thermal properties of eleven commercial unbranched saturated carboxylic esters. In this case, the esters proved to be interesting PCMs on accounts of their reliability. Similarly, Sari et al. [12] synthesized and characterized a series of stearic acid esters from butyl and isopropyl alcohol and glycerol, and proved that the esters produced were stable for up to 1000 cycles and had enthalpies of fusion between 121–151 J/g. While they show interesting properties, all esters listed above had phase change temperatures in the range between 20 and 60 °C, therefore proving to only be of interest for low to mid-temperature applications such as domestic hot water production, space heating and solar cooling [13,14,15,16,17]. 

Lactones are a particular class of esters widespread in nature and plants, in particular as biologically active compounds. They are cyclic esters that can form spontaneously via intramolecular reactions of hydroxy fatty acids, especially when the ring formed is five- or six-membered. Alternatively, lactones can be easily synthesized from their corresponding cyclic ketones through Baeyer-Villiger oxidation in the presence of a peroxyacid [18]. Lactones are extensively used in the food industry due to their ability to contribute to the aroma of various food and fruits (e.g., γ-decalactone is responsible for peach flavour) [19]. Additionally, they also find usage in other fields, for example, ε-caprolactone is currently extensively used as a precursor for the production of bio-based and biodegradable polyesters such as polycaprolactone (PCL) due to its suitable properties and natural occurrence. Similarly, γ-valerolactone can be obtained from cellulosic biomass and is currently being tested for the production of “green” fuels. All lactones can easily decompose due to the strain of the closed ring and the inclination of the cyclic ester bond to break to form more stable linear molecules [20]. For these reasons, lactones can be considered biodegradable as well as bio-based, hence holding great potential as possible bio-PCM. Moreover, differently from the fatty esters mentioned above, whose phase change occurs in low to mid temperature ranges, lactones are reported to possess a wide range of phase change temperatures from very low (e.g., −43 °C for γ-butyrolactone) to high (e.g., 150 °C for d-gluconic acid δ-lactone). Such temperature ranges would be interesting for various purposes. An important application could be, for example, industrial processes requiring high operational temperatures, which are responsible for more than 30% of the heat consumption of industrial process (e.g., food industry, paper industry, chemical industry). In general, process heat in industrial operations accounts for more than one third of the global total energy consumption, therefore finding sustainable solutions for the storage of process heat is essential [1,2,3,4,21,22]. Thus, bio-based and biodegradable lactones in these temperature ranges could become a more sustainable alternative to traditional systems. However, no studies have been reported in the literature for lactones as PCM, and little to no experimental thermal data on their phase change transitions and enthalpies of fusion is available.

In this study, five different lactones—γ-valerolactone, ε-caprolactone, (1*R*,3*r*,6*s*,8*S*)-4-oxatricyclo-[4.3.1.1^3,8^]undecan-5-one (abbreviated as oxa-adamantanone), 1,8,8-trimethyl-2-oxabicyclo[3.2.1]-octan-3-one (given name 1,2-campholide) and dibenzochromen-6-one (abbreviated DBC6O)—have been tested to assess their thermal properties and their suitability to act as PCMs for thermal energy storage applications. The first two were purchased commercially while the remaining three, which were not commercially available, were synthesized from cyclic natural ketones via Baeyer-Villiger oxidations. This is the first time that lactones have been examined for their suitability as PCM to the best of the authors’ knowledge. Additionally, this is the first time the thermal properties (phase change temperatures, degradation temperatures and enthalpies of fusion) of the lactones presented have been measured and reported in literature in any type of context. 

## 2. Results 

### 2.1. Structural Characterization of Lactones 

#### 2.1.1. ATR-IR

**Figure 1:**
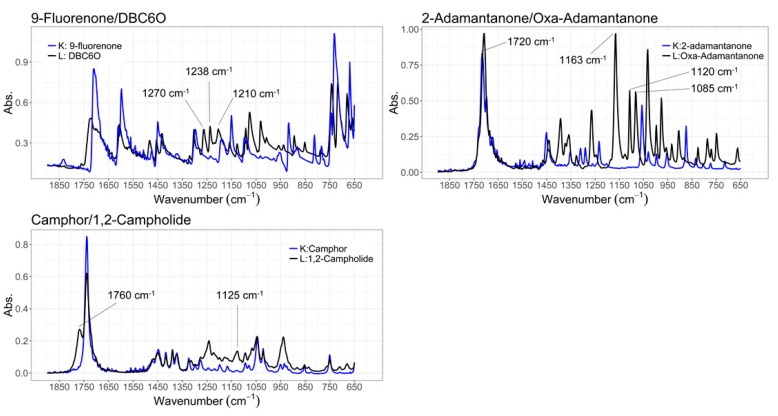
ATR-IR spectra of all lactones produced (in black, preceded by “L” in the legend) compared to the corresponding precursor cyclic ketones (in blue, preceded by “K” in the legend). The presence of the newly formed lactone bond was confirmed by the appearance of several peaks in the 1050–1270 cm^−1^ region from the C-O-C and C-O stretching in the ester bond. Additionally, a new band at 1760 cm^−1^ from the stretching of O-C=O in 1,2-campholide was visible.

In order to first confirm the effectiveness of the synthesis procedure reported by Olah et al. [23] and the formation of the desired products, the raw materials and lactones synthesized were fully characterized in terms of structural and purity analysis. As such, ATR-IR spectra of the lactones were registered and compared with those of the corresponding precursor cyclic ketone. Figure 1 shows the spectra recorded for each lactone compared to its starting cyclic ketone. Ketones are reported in blue and with a K preceding the given name in the legend, while lactones are in black and preceded by a letter L in the legend. 

As observed in all samples, the formation of the C-O-C ester bond is confirmed by the appearance of new peaks in the 1125–1275 cm^−1^ region. However, differences in the chemical structure give rise to shifts and variations in the spectra which enable the lactones to be identified. In the case of DBC6O, three peaks which are not present in the ketone spectra are now visible at 1270 cm^−1^, 1238 cm^−1^ and 1210 cm^−1^, and are the result of the stretching of the alkyl aryl C-O-C bond, the stretching of the vinyl C=C-O bond and the C-O stretching of the ester group, respectively. Concerning oxa-adamantanone, a sharp peak at 1163 cm^−1^ from the stretching of C-O from the ester group is visible, and a slight shift is observed in the peak from the main cyclohexanone’s structure at 1720 cm^−1^ to lower frequencies due to the addition of an additional oxygen in the ring and thus the formation of a seven-membered ring. Another two sharp peaks at 1120 and 1085 cm^−1^ are seen from the stretching of C-O in the aliphatic structure. In the spectra of 1,2-campholide, the presence of the lactone ring is confirmed by the appearance of a stretching band from O-C=O at 1760 cm^−1^ and one from the stretching of an aliphatic C-O bond at 1125 cm^−1^. 

#### 2.1.2. GC-MS

In order to further prove the formation of the produced lactones, the GC-MS analysis of the synthesized samples was performed. Table 1 shows the retention times retrieved and the main fragmentation peaks observed along with the corresponding relative intensities. No side peaks from volatile impurities were detected in the GC chromatograms of oxa-adamantanone and DBC6O. On the other hand, a side peak of about 5% intensity could be noticed at 7.45 min for 1,2-campholide. Overall, the MS reports for all three lactones matched with the relative spectra in the official National Institute of Standards and Technology (NIST) database library and with the fragmentation peaks reported by Olah et al. [23], which confirmed that the synthesis was successful. Generally, lactones show a characteristic breakage of the O-C=O lactone group leaving as CO_2_ corresponding to a loss of 44 *m*/*z* [24]. Such fragmentation can only occur in lactones and not in their precursor cyclic ketones due to the presence of an additional oxygen. This was observed in oxa-adamantanone at 122 *m*/*z*, which corresponds to the loss of 44 *m/z* from the molecular ion at 166 *m*/*z* which is not visible. In 1,2-campholide a fragment due to loss of CO_2_ from the molecular ion at 168 *m/z*, is visible at 124 *m*/*z*. Unlike oxa-adamantanone and 1,2-campholide, DBC6O forms two different fragments at 168 and 139 *m*/*z*, each corresponding to the loss of 28 *m*/*z* from the molecular ion (196 *m*/*z*) and the previous fragment (168 *m*/*z*), respectively. This is due to loss of CO twice instead of CO_2_ once, supposedly caused by the higher stability of the aromatic-aliphatic structure. Therefore, the formation of the lactone group was confirmed. Additionally, the typical breakdown of the aliphatic chain due to progressive loss of –CH_2_ (14 *m*/*z*) or CH_2_–CH_2_ (28 *m*/*z*) was observed for all samples, as similarly stated for fatty esters by Ravotti et al. [9,10]. 

### 2.2. Analysis of Thermal Properties

After successfully confirming the structures of the lactones produced and the validity of the synthesis procedure, the thermal properties of both the synthesized and the commercially purchased substances were investigated. Table 2 below summarizes the thermal properties retrieved for each compound. In the case of oxa-adamantanone and DB6CO only one phase change transition peak was observed, which implies the absence of non-volatile impurities in a significant amount within the sample. Therefore, in addition to the lack of volatile impurities in the GC chromatogram, high purities for oxa-adamantanone and DBC6O could be assumed. No side peaks in the DSC could be noticed for 1,2-campholide, however due to inconsistent crystallization, the presence of low-percentage non-volatile impurities could not be completely excluded. Therefore, no certain conclusions on the purity of 1,2-campholide could be drawn. 

**Figure 2:**
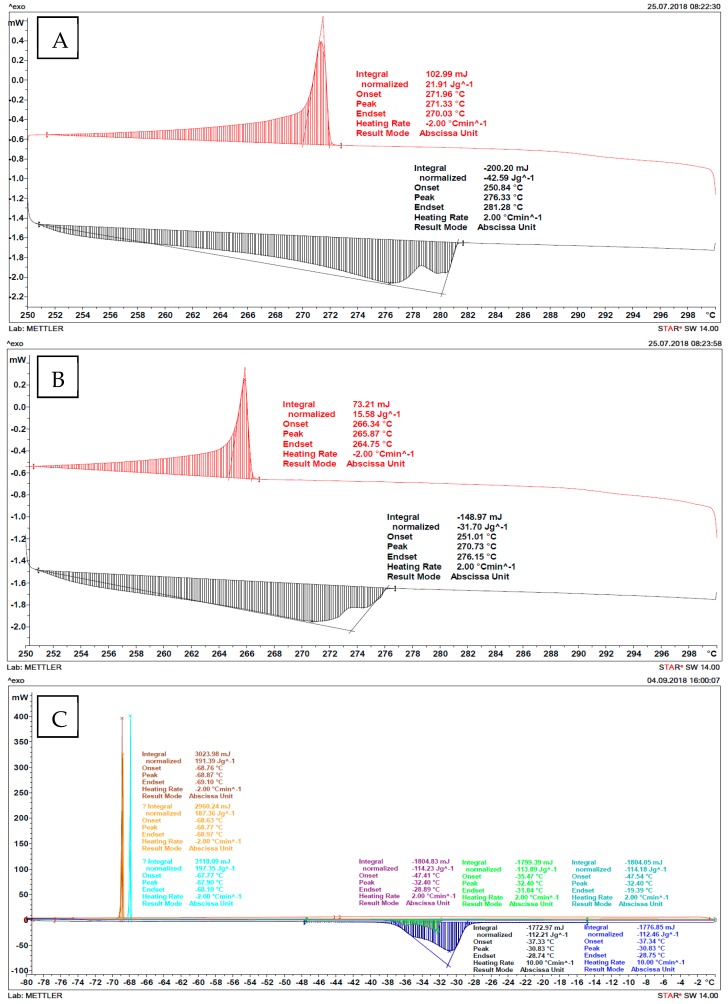
(**A**,**B**) DSC graphs of the 5th and 6th heating-cooling cycles of oxa-adamantanone at 2 °C/min respectively. The enthalpies of each cycle can be seen progressively decreasing to about 15 J/g. The phase change transition of oxa-adamantanone occurs over a broad temperature range of 30 °C. (**C**) DSC measurement of γ-valerolactone with six consecutive heating-cooling cycles at 10 °C/min and 2 °C/min for comparison with oxa-adamantanone results. Here although no crystallization took place for 10 °C/min heating rates, the enthalpies of fusion were stable for all other cycles and the phase transition was registered over narrow temperature ranges.

As can be observed, the lactones present onset melting temperatures falling in a range from −40 °C for γ-valerolactone to 290 °C for oxa-adamantanone. Various degrees of supercooling from 10 °C to 30 °C were observed in the DSC for all samples with the exception of oxa-adamantanone which displayed a 5 °C difference between the onset of melting and the onset of crystallization. All lactones produced were characterized by low enthalpies of fusion below 70 J/g, with only the commercial ε-caprolactone and γ-valerolactone presenting values above 100 J/g. 1,2-Campholide showed a general instability to six continuous heating and cooling cycles with a decreasing enthalpy of fusion after each cycle or no crystallization taking place at all after the first cycle. Oxa-adamantanone presented reproducible crystallization, but like the aforementioned lactone, its enthalpy of fusion showed decreasing values of 40 J/g to 15 J/g by the sixth cycle. For this reason, the average enthalpies of fusion calculated for oxa-adamantanone and 1,2-campholide have been starred in Table 2 to signal potentially incorrect values due to the suspected degradation of the materials taking place in the same temperature interval as the phase change. Figure 2A,B shows the DSC graphs measured at 2 °C/min from the 5th and 6th heating and cooling cycle of oxa-adamantanone. Here, the progressive decrease of the phase change enthalpies at each phase change is visible. Additionally, oxa-adamantanone and 1,2-campholide were both characterized by phase changes occurring over a broad temperature range for both heating rates, for example with onset melting around 250 °C and endset at 280 °C for oxa-adamantanone, and onset around 80 °C and endset at 100 °C for 1,2-campholide. On the contrary, all the other lactones presented narrow phase change peaks. Figure 2C shows the DSC retrieved from all heating and cooling cycle of γ-valerolactone at both 10 °C/min and 2 °C/min for comparison with oxa-adamantanone measurements. In the case of γ-valerolactone, no crystallization was observed at 10 °C/min, but the enthalpy of fusion was constant for the cycles where crystallization occurred and the phase change transitions were over narrow temperature ranges. Early starting degradation temperatures were noted for all samples from 90–100 °C, with all molecules being completely degraded by 200–310 °C.

## 3. Discussion

**Figure 3:**
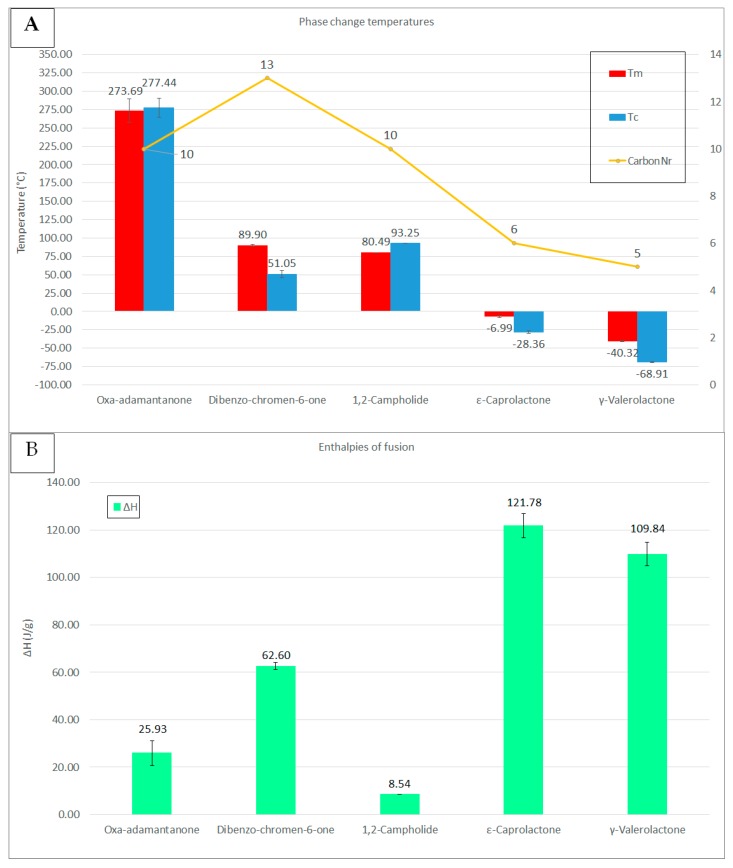
(**A**) Melting (red bars, Tm) and crystallization (blue bars, Tc) onset phase change temperatures in °C measured for both the commercial and synthesized lactones reported in this study. The carbon number of each lactone (yellow line) is showed to observe possible trends correlating the chemical structure to the phase change transitions. In general, no clear correlation between the carbon number and phase change temperatures was noticed. In the case of 1,2-campholide, no error bars could be reported due to inconsistent crystallization. (**B**) Enthalpies of fusion in J/g (green bars, ΔH) for the lactones described previously. Analogously to what explained above, the error bars for 1,2-campholide’s enthalpy could not be calculated. All synthesized lactones show enthalpies of fusion ≤ 70 J/g while commercial ones are above 100 J/g. Oxa-adamantanone’s enthalpy has a higher standard deviation due to the phase change occurring in the same temperature range as the thermal degradation, thus causing the enthalpy to greatly decrease after each heating-cooling cycle.

The lactones presented herein show a promising range of phase change temperatures, which would be valuable for several applications. The thermal properties of the lactones presented in this study, namely phase change temperatures and enthalpies of fusion, compared to the carbon number of each molecule are shown in Figure 3. This far, only salt hydrates or eutectic salt/water mixtures have been reported to possess melting temperatures in the sub-zero region (from −1 °C down to even −100 °C). Therefore, bio-lactones with a melting point in such a region would be an interesting alternative to inorganic PCMs due to the advantages described in the previous pages. As reported by Li et al. [14], possible areas of applications for sub-zero PCMs include cold air distribution and cold storage transportation (−5 °C to −10 °C), frozen food transportation and preservation, concrete isolation in cold locations (−10 °C to −35 °C) and stabilization of temperature fluctuation in freezers (−30 °C to −60 °C). Concerning temperatures in the range 80 °C to 400 °C, only a few studies involving PCMs have been reported, mostly dedicated to applications in industrial heating and improvement of the energy efficiency in power plants. Typically, the PCMs used for such tests comprise nitrates, carbonates, chlorides and fluorides [15,16,17]. Therefore, bio-based organic PCM in this temperature range would also pose a valuable alternative. However, although the lactones presented interesting phase change transition temperatures, they also exhibited several problems. 1,2-Campholide and oxa-adamantanone showed an unreliable behaviour with no crystallization occurring at all for some cycles or experiments and with a progressively decreasing enthalpy of fusion over each cycle. This is thought to be due to decomposition of the material starting in the same temperature range as the phase change transition. For this reason, the average enthalpies of fusion calculated are considered inconclusive and potentially incorrect, as part of the material might have already degraded during the first cycle prior to the phase change occurring. In general, with respect to the enthalpies of fusion, only commercial ε-caprolactone and γ-valerolactone reported mild ΔH values around 110–120 J/g, while all other samples showed values below 60 J/g, which defies the purpose of using such materials as PCM. Additionally, as mentioned above, all samples including the commercial ones, demonstrated high supercooling degrees with only oxa-adamantanone undergoing supercooling below 5 °C. While such behaviour is thought to be enhanced by the small scale of the sample used for DSC analysis, supercooling is not usually observed for organic materials such as paraffins or fatty esters under the same measurement conditions [5,6,7,8,9,10]. On the other hand, it is a well-known problem found in salt hydrates which is usually solved by adding a nucleating agent having similar chemical and thermal properties to the substance, with the aim to promote nucleation and crystal growth [5,6,7]. Apart from the supercooling, DBC6O, γ-valerolactone and ε-caprolactone were thermally stable over six heating and cooling cycles at different heating rates. By comparing both phase change temperatures and enthalpies of fusion to the carbon number of the molecule, no clear trend is revealed. The commercial lactones ε-caprolactone and δ-valerolactone possess markedly lower temperatures and carbon number compared to the synthesized lactones. This seemed to imply that a lower carbon number causes the phase change to occur at lower temperatures. However, both oxa-adamantanone and 1,2-campholide have the same carbon number (10) but a difference between their onset melting temperatures of 193 °C. On the other hand, despite the presence of aromatic rings in the structure which could give rise to π-stacking and thus a more tightly packed crystalline structure, and the highest carbon number amongst the lactones, DBC6O has an onset melting temperature comparable to that of 1,2-campholide. Similarly to what observed for phase change temperatures, no clear relation between the values of enthalpy of fusion and the carbon number or chemical structure could be deduced. Therefore, no conclusions on possible trends correlating the chemical structure to the thermal properties could be drawn. 

## 4. Materials and Methods

### 4.1. Materials

2-Adamantanone (≥90%), camphor (≥99%) and 9-fluorenone (≥99%) were used as synthesis precursors. Sodium percarbonate (Na_2_CO_3_ ·1.5 H_2_O_2_, ≥99%) and trifluoroacetic acid (TFA, CF_3_CO_2_H, ≥99%) were used to form in-situ trifluoroperoxyacetic acid (TFPAA, CF_3_CO_3_H) which served to oxidize the cyclic ketones to lactones through Baeyer Villiger oxidation. Deionized water and diethyl ether (Et_2_O, ≥99%) were used to quench the reaction mixture and separate the resulting lactone respectively, while sodium sulfate anhydrous (Na_2_SO_4_, ≥99%) was used as a water-absorbing agent for the elimination of water. ε-Caprolactone (≥99%) and γ-valerolactone (≥99%) were tested without further treatment. All chemicals listed above were purchased from Sigma Aldrich (St. Louis, MI, USA) and used without any prior purification. The chemical structures, IUPAC names, given names and abbreviations of both the lactones and their ketone precursors are reported in Appendix A.

### 4.2. Synthesis of Lactones

**Scheme 1:**
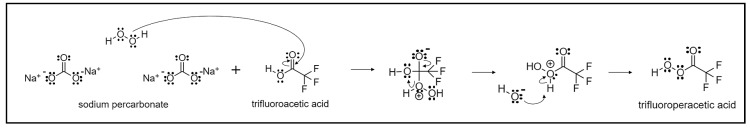
The mechanism behind the in-situ formation of peroxytrifluoroacetic acid (or trifluoroperacetic acid). The hydrogen peroxide contained in sodium percarbonate acts as a nucleophilic and attacks the carbonyl trifluoroacetic acid to form trifluoroperacetic acid and water as a leaving group.

As mentioned above, lactones that could not be purchased commercially were synthesized in-house. The synthesis of lactones was performed according to the method proposed by Olah et al. [23]. The Baeyer-Villiger oxidation is a standard synthesis procedure for producing esters from ketones, which comprises of using a peroxyacid as an oxidizing agent. While the oxidizing agent used is generally m-chloroperoxybenzoic acid, it might pose some risks to safety as it is shock sensitive and may cause fire upon contact with flammable material. As such, a safer alternative used here is peroxytrifluoroacetic acid (or trifluoroperacetic acid), which can be easily generated in situ from sodium percarbonate with trifluoroacetic acid. The mechanism involving the in-situ formation of peroxytrifluoroacetic acid from sodium percarbonate is shown in Scheme 1. 

**Scheme 2:**
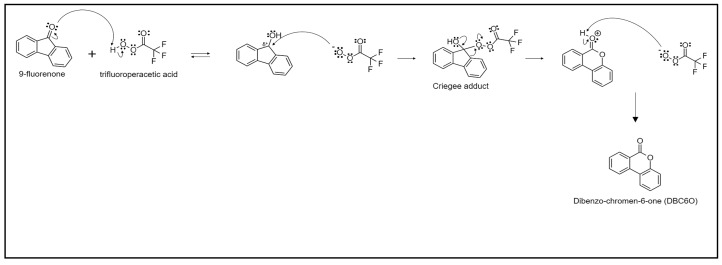
Scheme summarizing the mechanism of Baeyer Villiger oxidation to form lactones from cyclic ketones. The oxygen on the ketone gets protonated by the peroxy acid thus causing the negatively charged acid to attack the carbonyl. The lactone is then created through a concerted mechanism via Criegee adduct, which then sees trifluoroperacetic acid acting as a leaving group.

In summary, the hydrogen peroxide in sodium percarbonate acts as a nucleophilic and attacks the carbonyl of trifluoroacetic acid hence causing the hydroxyl group to act as a leaving group and to form water and trifluoroperacetic acid through deprotonation. As reported by Kjonaas et al. [25] peroxytrifluoroacetic acid can also be formed from readily available household cleaning agents containing sodium percarbonate (e.g., most washing machine powders) and is therefore also a more accessible alternative to *m*-chloroperoxybenzoic acid. In the presence of cyclic ketones, cyclic esters (namely lactones) can be formed with the inclusion of an additional oxygen to the closed ring in the α position with respect to the carbonyl group. This means, for example, that in the case of five- membered cyclic ketones, due to the addition of an extra carbon-oxygen bond in the structure a six-membered ring will be formed instead. The reaction mechanism is described extensively by Alvarez-Idaboy [26], Krow [27] and Kjonaas [25], and is shown schematically in Scheme 2 with the synthesis of dibenzo-chromen-6-one as an example. 

The oxygen of the carboxylic group is first protonated by the peroxy acid thus forming a partial positive charge on the carbonyl. The deprotonated peroxytrifluoroacetic acid attacks then the carbonyl to create an instable adduct commonly referred to as Criegee adduct. Here, the double bond between the oxygen and carbon of the carbonyl group is newly formed therefore pushing the group in α to the carbonyl to start a concerted step. This results in a new oxygen atom being included in the closed ring of the ketone, which is now a new ester group, and in peroxytrifluoroacetic acid acting as a leaving group. In summary, a 25 mL two-necked round bottom flask with a magnetic stirrer topped with a reflux condenser was cooled down to 0 °C by means of an ice bath. Afterwards, 3.3 mmol of cyclic ketone were added to the flask together with 6.7 mL of TFA and left to dissolve completely while stirring at 500 rpm. Following this, 6.7 mmol of sodium percarbonate were slowly added to the reaction mixture over a period of 40 min to form in situ peroxytrifluoroacetic acid. The addition was performed by inserting every 5 min some milligrams of sodium percarbonate in the round bottom flask by means of a metal spatula. Upon completion of the addition of sodium percarbonate, the mixture was brought to room temperature and stirred for 2 h for camphor, and overnight for 9-fluorenone and 2-adamantanone. The mixture was then quenched with 14 mL of deionized water, and the lactone produced was separated from unreacted reagents with three consecutive washings of diethyl ether and deionized water in a separating funnel. Subsequently, the organic phase was washed with a saturated solution of Na_2_CO_3_ in deionized water three times. Lastly, the product was dried first with Na_2_SO_4_ for 10 min followed by the evaporation of the solvent using the rotary evaporator at 240 mbar for 30 min and a water bath at 40 °C. As previously done by Ravotti et al. [9,10], all syntheses were repeated three times to ensure reproducibility. Therefore, each ester was analyzed three times.

### 4.3. Analytical Instruments and Methods

#### 4.3.1. Attenuated Total Reflectance Infrared Spectroscopy (ATR-IR)

In order to gain structural information and confirm the formation of the lactone group, ATR-IR spectroscopy (Cary 630 instrument by Agilent Technologies, Santa Clara, CA, USA) in the wavenumber range between 4000 and 600 cm^−1^ with 4 cm^−1^ resolution was used. No prior sample preparation was needed, and a background spectra was registered every 30 min. Both sample and background spectrum were registered with 32 scans.

#### 4.3.2. Gas Chromatography coupled with Mass Spectroscopy (GC-MS) 

Gas chromatograms were measured on a Clarus 590 GC instrument (Perkin Elmer, Waltham, MA, USA) connected to a Perkin Elmer MS Clarus SQ 8 S with EI standard source, with 4.0 mm Glass inlet liner with deactivated Wool in split mode 1:50 on a Perkin Elmer Elite 5 30 m × 250 μm × 0.25 μm column and 1.0 mL/min flow rate. The oven program was 100 °C for 2 min, then 10 °C/min heating rate to 300 °C and afterwards hold for 5 min. The temperature of the MSD transfer line was 250 °C. Mass spectra were measured with electron ionization (EI) at 70 eV and source temperature of 200 °C. The instrument was scanned between *m*/*z* 50 and 500 at a scan time of 0.3 s and inter-scan delay of 0.04 s. Perfluoroterbutylamine (PFTBA, Sigma Aldrich) served for tuning of the MS. The samples were diluted in chloroform (≥99.9% GC grade, Sigma Aldrich) with a concentration of 0.1 mg/mL and the injection volume was 1 μL.

#### 4.3.3. Differential Scanning Calorimetry (DSC)

Analysis of melting and crystallization temperatures was conducted via DSC 823^e^ by Mettler Toledo (Columbus, OH, USA). Three consecutive heating-cooling cycles at both 2 °C/min and 10 °C/min heating rates for all samples were measured to test the reproducibility of the samples. The samples were measured under a constant flow of nitrogen at a rate of 100 mL/min, and sample masses were between 5 mg and 20 mg. The DSC was calibrated with an indium standard before the measurements, and the uncertainty of the instrument was ±0.1 K. A prior screening measurement was performed with two heating-cooling cycles at 10 °C/min between −50 °C and 300 °C for all samples in order to locate the phase change transition. Afterwards, appropriate temperature measuring ranges were selected for each lactone based on the results of the screening test. Generally, measuring ranges were defined as the phase transition ranges ±20 °C from both onset melting and crystallization sides. The degree of supercooling was estimated considering the difference between the onset melting peak and the onset crystallization peak reported from the instrument, keeping in consideration that the DSC indicates a strong tendency for the samples to supercool. The values reported have been calculated as follows: for each replicate of the same lactone, the averages for all six cycles at 10 °C/min and 2 °C/min for T_m_, T_c_, ΔH and T_degradation_ were derived. Afterwards, the average of the three final values obtained for each lactone repetition was retrieved and is reported in the following pages alongside with the relative error. Therefore, all uncertainty values reported in the subsequent pages are based on replicate measurements, therefore denoting the accuracy of the results. The results were evaluated on Mettler Toledo STAR^e^ Version 14 software.

#### 4.3.4. Thermal Gravimetric Analysis (TGA)

The samples’ thermal stability and degradation were analyzed by TGA on a STAR^e^ 2 System by Mettler Toledo in the temperature range between 25 °C and 600 °C with a heating rate of 10 °C/min and sample masses between 15–20 mg. The uncertainty of the instrument’s balance is reported to be 0.1 μg. For each sample, a blank measurement of the empty crucible was performed with the same method as described above for correct baseline subtraction. The starting degradation temperature was typically defined as the earliest temperature for which mass losses ≥ 5% were observed. Similarly, the end temperature degradation was marked as the earliest temperature for which the mass loss was ≥99%. The results were evaluated on Mettler Toledo STAR^e^ Version 14 software.

## 5. Conclusions and Outlook

In the presented study, three synthesized lactones shortened as oxa-adamantanone, DBC6O and 1,2-campholide, and two commercial ones, ε-caprolactone and γ-valerolactone, have been tested as possible bio-based and biodegradable PCMs, and their thermal properties have been evaluated and reported for the first time in literature to the best of the authors’ knowledge. The lactone syntheses were replicated three times each to ensure the reproducibility of the Baeyer-Villiger oxidation of the corresponding cyclic ketones with peroxytrifluoroacetic acid generated in situ from sodium percarbonate and trifluoroacetic acid. Firstly, the synthesis procedure was validated and the structures of the desired synthesized lactones were confirmed by means of structural analysis through GC-MS and ATR-IR. Both techniques confirmed a high degree of purity for DBC6O and oxa-adamantanone and allowed to distinguish between each particular structure. Following structural analysis, the lactones were tested in DSC and TGA in order to gain information on phase change temperatures, temperatures of degradation, enthalpies of fusion and cycling stability. In summary, the lactones present a broad range of phase change temperatures extending from −40 °C for γ-valerolactone to 290 °C for oxa-adamantanone. Such temperatures are nowadays being covered in LHS applications mainly by salt hydrates or other inorganic PCM, and no mention of organic PCMs at such high or low temperatures has been reported in the literature. Hence, the lactones show interesting melting temperatures with great potential for several applications, including frozen food transportation and industrial heating. However, despite their potential, all lactones display early degradation and general thermal instability. In particular, oxa-adamantanone and 1,2-campholide melt in the range of their thermal degradation over broad temperature ranges causing a decrease in the enthalpy of fusion and a high unreliability with difficulties to crystallize. Conversely, DBC6O, γ-valerolactone and ε-caprolactone appear to be thermally stable over consecutive cycles. Of all the lactones shown hereby, only oxa-adamantanone is characterized by supercooling ≤5 °C, while for the others supercooling between 10 °C and 30 °C is observed. High degrees of supercooling are undesirable as they do not assure a proper functioning of the LHS since the randomness of such phenomena does not allow to extract heat within a reproducible temperature interval. While the phase change temperatures of the lactones are of interest, all synthesized lactones possess very low energy densities ≤60 J/g, which highlight their unsuitability as PCMs. In order to be considered valuable as new PCMs, the compounds should be characterized by the highest values of heat of fusion possible, or at least equivalent to competing materials such as for example paraffins or salt hydrates. Both paraffins and salt hydrates typically show values between 150–250 J/g, therefore lactones fail to provide the energy density necessary to satisfy LHS requirements [5,6,7]. While ε-caprolactone and γ-valerolactone report slightly higher ΔH values of 110–120 J/g, such values in addition to the problems of supercooling mentioned above challenges their competitiveness with salt hydrates. In fact, although organic PCMs in general present higher prices compared to salt hydrates, owing to the advantages mentioned in the previous pages (e.g., no segregation, no supercooling, no corrosivity) they would remain competitive alternatives to salt hydrates. However, in order to fully determine whether lactones’ supercooling is comparable to salt hydrates, tests in larger setups should be conducted first. In conclusion, while the lactones presented show a wide range of phase transition temperatures from subzero to above 200 °C, they proved to be unfit as PCM due to unreliable thermal stability, high tendency to supercooling and low enthalpies of fusion. As this study is part of a broader investigation conducted by the authors on esters as new bio-based PCM, further studies will be carried out on other classes of esters, such as diesters and triglycerides. 

## Figures and Tables

**Table 1:** Retention times measured on a Supelco SLB-5ms GC column and fragmentation patterns reported from MS spectra with relative intensities in brackets. Generally, the molecular ion was visible for 1,2-campholide and DBC6O, but not for oxa-adamantanone. Oxa-adamantanone and 1,2-campholide showed the characteristic loss of 44 *m*/*z* corresponding to loss of CO_2_ fragment. In DBC6O two losses of CO (28 *m*/*z*) instead of CO_2_ were observed, possibly due to the higher stability of the aliphatic-aromatic structure. Therefore, the formation of the ester group was proved. Additionally, consecutive breakage of the aliphatic ring was visible by loss of CH_2_ fragments (14 *m*/*z*).

Compound	Retention Time (min)	Fragmentation Peaks, *m*/*z* with Relative Intensities (%)
**Oxa-adamantanone**	9.27	122 (9), 94 (9), 80 (100), 67 (10), 55 (4)
**1,2-Campholide**	5.87	168 (5), 125 (83), 111 (78), 97 (6), 86 (5), 71 (5), 55 (100)
**DBC6O**	12.81	196 (100), 168 (27), 139 (23), 113 (2), 98 (2), 84 (2), 70 (9), 55 (1)

**Table 2:** Summary of thermal properties registered for both the commercial and the synthesized lactones presented in this study. The values and the errors reported are calculated based on the averages and standard deviations respectively registered in all six measurements and three repetitions. A wide range of temperatures was observed, however high degrees of supercooling and low enthalpies of fusion below 120 J/g marked the lactones’ unsuitability as PCM. 1,2-Campholide showed inconsistent crystallization, with several cycles lacking any clear crystallization peak. As such, the errors could not be derived and are here reported as N.A (non-applicable). Given that oxa-adamantanone and 1,2-campholide’s phase change occurs within the same range of their thermal decomposition, the values of average enthalpy calculated have been starred to signal a potentially inaccurate and inconclusive value.

Compound	Tc (Peak, °C)	Tm (peak, °C)	Tc (Onset, °C)	Tm (onset, °C)	ΔH (J/g)	T (°C)
Oxa-adamantanone	283.36 ± 7.97	287.69 ± 7.27	284.31 ± 7.95	281.82 ± 11.46	25.93 * ± 5.27	150 ± 10–250 ± 10
1,2-Campholide	91.71 ± N.A.	95.43 ± N.A.	93.25 ± N.A.	80.49 ± N.A.	8.54 *± N.A.	93 ± 6–225 ± 13
DBC6O	49.80 ± 5.20	92.83 ± 0.30	51.05 ± 4.94	89.90 ± 1.34	62.60 ± 1.46	175 ± 5–307 ± 21
ε-Caprolactone	−29.63 ± 1.50	−1.55 ± 1.77	−28.36 ± 1.66	−6.99 ± 1.28	121.7 ± 5.04	105 ± 7–205 ± 7
γ-Valerolactone	−69.03 ± 0.74	−32.13 ± 0.50	−68.91 ± 0.73	−40.32 ± 0.99	109.8 ± 5.03	93 ± 4–188 ± 4

**Figure A1:**
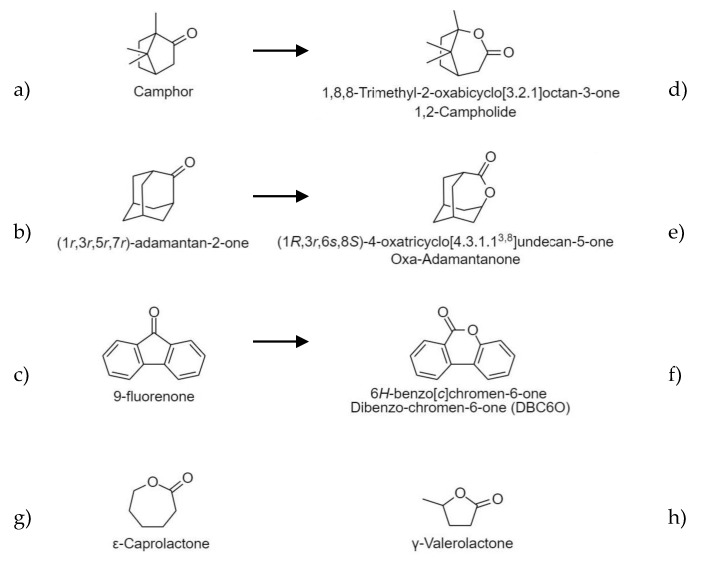
Structures, IUPAC and given names of precursor cyclic ketones (**a**,**b**,**c**), the relative lactones produced (**d**,**e**,**f**) and the commercial lactones studied (**g**,**h**).

## List of Abbreviations

**ATR-IR**	Attenuated Total Reflectance InfraRed Spectroscopy
**DBC6O**	Dibenzo-chromen-6-one
**DSC**	Differential Scanning Calorimetry
**Et_2_O**	Diethyl Ether
**GC-MS**	Gas-Chromatography coupled with Mass Spectrometry
**H_2_O_2_**	Hydrogen Peroxide
**LHS**	Latent Heat Storage
**Na_2_CO_3_**	Sodium Carbonate
**Na_2_SO_4_**	Sodium sulfate
**NIST**	National Institute of Standards and Technology
**PCL**	Polycaprolactone
**PCM**	Phase Change Material
**PFTBA**	Perfluoroterbutylamine
**SNSF**	Swiss National Science Foundation
**T_c_**	Crystallization Temperature
**T_m_**	Melting Temperature
**TFA**	Trifluoroacetic acid
**TFPAA**	Trifluoroperoxyacetic acid
**TGA**	Thermogravimetric Analysis
**ΔH**	Enthalpy of fusion
